# FANTOM5 CAGE profiles of human and mouse samples

**DOI:** 10.1038/sdata.2017.112

**Published:** 2017-08-29

**Authors:** Shuhei Noguchi, Takahiro Arakawa, Shiro Fukuda, Masaaki Furuno, Akira Hasegawa, Fumi Hori, Sachi Ishikawa-Kato, Kaoru Kaida, Ai Kaiho, Mutsumi Kanamori-Katayama, Tsugumi Kawashima, Miki Kojima, Atsutaka Kubosaki, Ri-ichiroh Manabe, Mitsuyoshi Murata, Sayaka Nagao-Sato, Kenichi Nakazato, Noriko Ninomiya, Hiromi Nishiyori-Sueki, Shohei Noma, Eri Saijyo, Akiko Saka, Mizuho Sakai, Christophe Simon, Naoko Suzuki, Michihira Tagami, Shoko Watanabe, Shigehiro Yoshida, Peter Arner, Richard A. Axton, Magda Babina, J. Kenneth Baillie, Timothy C. Barnett, Anthony G. Beckhouse, Antje Blumenthal, Beatrice Bodega, Alessandro Bonetti, James Briggs, Frank Brombacher, Ailsa J. Carlisle, Hans C. Clevers, Carrie A. Davis, Michael Detmar, Taeko Dohi, Albert S.B. Edge, Matthias Edinger, Anna Ehrlund, Karl Ekwall, Mitsuhiro Endoh, Hideki Enomoto, Afsaneh Eslami, Michela Fagiolini, Lynsey Fairbairn, Mary C. Farach-Carson, Geoffrey J. Faulkner, Carmelo Ferrai, Malcolm E. Fisher, Lesley M. Forrester, Rie Fujita, Jun-ichi Furusawa, Teunis B. Geijtenbeek, Thomas Gingeras, Daniel Goldowitz, Sven Guhl, Reto Guler, Stefano Gustincich, Thomas J. Ha, Masahide Hamaguchi, Mitsuko Hara, Yuki Hasegawa, Meenhard Herlyn, Peter Heutink, Kelly J. Hitchens, David A. Hume, Tomokatsu Ikawa, Yuri Ishizu, Chieko Kai, Hiroshi Kawamoto, Yuki I. Kawamura, Judith S. Kempfle, Tony J. Kenna, Juha Kere, Levon M. Khachigian, Toshio Kitamura, Sarah Klein, S. Peter Klinken, Alan J. Knox, Soichi Kojima, Haruhiko Koseki, Shigeo Koyasu, Weonju Lee, Andreas Lennartsson, Alan Mackay-sim, Niklas Mejhert, Yosuke Mizuno, Hiromasa Morikawa, Mitsuru Morimoto, Kazuyo Moro, Kelly J. Morris, Hozumi Motohashi, Christine L. Mummery, Yutaka Nakachi, Fumio Nakahara, Toshiyuki Nakamura, Yukio Nakamura, Tadasuke Nozaki, Soichi Ogishima, Naganari Ohkura, Hiroshi Ohno, Mitsuhiro Ohshima, Mariko Okada-Hatakeyama, Yasushi Okazaki, Valerio Orlando, Dmitry A. Ovchinnikov, Robert Passier, Margaret Patrikakis, Ana Pombo, Swati Pradhan-Bhatt, Xian-Yang Qin, Michael Rehli, Patrizia Rizzu, Sugata Roy, Antti Sajantila, Shimon Sakaguchi, Hiroki Sato, Hironori Satoh, Suzana Savvi, Alka Saxena, Christian Schmidl, Claudio Schneider, Gundula G. Schulze-Tanzil, Anita Schwegmann, Guojun Sheng, Jay W. Shin, Daisuke Sugiyama, Takaaki Sugiyama, Kim M. Summers, Naoko Takahashi, Jun Takai, Hiroshi Tanaka, Hideki Tatsukawa, Andru Tomoiu, Hiroo Toyoda, Marc van de Wetering, Linda M. van den Berg, Roberto Verardo, Dipti Vijayan, Christine A. Wells, Louise N. Winteringham, Ernst Wolvetang, Yoko Yamaguchi, Masayuki Yamamoto, Chiyo Yanagi-Mizuochi, Misako Yoneda, Yohei Yonekura, Peter G. Zhang, Silvia Zucchelli, Imad Abugessaisa, Erik Arner, Jayson Harshbarger, Atsushi Kondo, Timo Lassmann, Marina Lizio, Serkan Sahin, Thierry Sengstag, Jessica Severin, Hisashi Shimoji, Masanori Suzuki, Harukazu Suzuki, Jun Kawai, Naoto Kondo, Masayoshi Itoh, Carsten O. Daub, Takeya Kasukawa, Hideya Kawaji, Piero Carninci, Alistair R.R. Forrest, Yoshihide Hayashizaki

**Affiliations:** 1 Division of Genomic Technologies, RIKEN Center for Life Science Technologies, Yokohama, Kanagawa 230-0045, Japan; 2 RIKEN Omics Science Center, Yokohama, Kanagawa 230-0045, Japan; 3 Department of Medicine, Karolinska Institutet, 141 86, Stockholm, Sweden; 4 Karolinska University Hospital, Center for Metabolism and Endocrinology, 141 86, Stockholm, Sweden; 5 Scottish Centre for Regenerative Medicine, University of Edinburgh, 5 Little France Drive, Edinburgh EH16 4UU, UK; 6 Department of Dermatology and Allergy, Charite University Medicine Berlin, Charitéplatz 1, 10117 Berlin, German; 7 The Roslin Institute and Royal (Dick) School of Veterinary Studies, University of Edinburgh, Edinburgh, Midlothian EH25 9RG, UK; 8 Australian Infectious Diseases Research Centre, The University of Queensland, St Lucia, QLD 4072, Australia; 9 School of Chemistry and Molecular Biosciences, The University of Queensland, St Lucia, QLD 4072, Australia; 10 Bio-Rad Laboratories Pty Ltd, Hercules, California 94547, USA; 11 The University of Queensland Diamantina Institute, The University of Queensland, Woolloongabba, QLD 4102 Australia; 12 IRCCS Fondazione Santa Lucia, Via del Fosso di Fiorano 64, 00143 Rome, Italy; 13 Australian Institute for Bioengineering and Nanotechnology (AIBN), University of Queensland, Brisbane, St Lucia, QLD 4072, Australia; 14 Division of Immunology, Institute of Infectious Diseases and Molecular Medicine (IDM), University of Cape Town, Anzio Road, Observatory 7925, Cape Town, South Africa; 15 Immunology of Infectious Diseases, Faculty of Health Sciences, South African Medical Research Council (SAMRC), University of Cape Town, Anzio Road, Observatory 7925, Cape Town, South Africa; 16 International Centre for Genetic Engineering and Biotechnology, Cape Town Component, Anzio Road, Observatory 7925, Cape Town, South Africa; 17 Hubrecht Institute, Royal Netherlands Academy of Arts and Sciences, Uppsalalaan 8, 3584 CT Utrecht, The Netherlands; 18 University Medical Centre Utrecht, Postbus 85500, 3508 GA Utrecht, The Netherlands; 19 Genomics, Cold Spring Harbor Laboratory, Cold Spring Harbor, New York 11797, USA; 20 Institute of Pharmaceutical Sciences, ETH Zurich, Vladimir-Prelog-Weg 3, HCI H 303, 8093 Zurich, Switzerland; 21 Gastroenterology, Research Center for Hepatitis and Immunology, Research Institute National Center for Global Health and Medicine, Ichikawa, Chiba 272-8516, Japan; 22 Department of Otology and Laryngology, Harvard Medical School, Boston, Massachusetts 02114, USA; 23 Department of Internal Medicine III, University Hospital Regensburg, F.-J.-Strauss Allee 11, D-93053 Regensburg, Germany; 24 RCI Regensburg Centre for Interventional Immunology, University Hospital Regensburg, F.-J.-Strauss Allee 11, D-93053 Regensburg, Germany; 25 Department of Biosciences and Nutrition, Karolinska Institutet, Halsovagen 7-9, SE-141 83 Huddinge, Sweden; 26 RIKEN Center for Integrative Medical Sciences, Yokohama, Kanagawa 230-0045, Japan; 27 Laboratory for Neuronal Differentiation and Regeneration, RIKEN Center for Developmental Biology, Chuou-ku, Kobe 650-0047, Japan; 28 Department of Bioinformatics, Medical Research Institute, Tokyo Medical and Dental University, Bunkyo-ku, Tokyo 113-8510, Japan; 29 F.M. Kirby Neurobiology Center, Children's Hospital, Harvard Medical School, Boston, Massachusetts 02115, USA; 30 The University of Texas Health Science Center at Houston, Houston, TX 77251-1892, USA; 31 Cancer Biology Program, Mater Medical Research Institute, South Brisbane, Queensland 4101, Australia; 32 Berlin Institute for Medical Systems Biology, Max Delbrueck Center, Robert Roessle Str.10, 13125 Berlin, Germany; 33 Department of Medical Biochemistry, Tohoku University Graduate School of Medicine, Sendai, Miyagi 980-8575, Japan; 34 Experimental Immunology, Academic Medical Center, University of Amsterdam, Meibergdreef 9, 1105 AZ Amsterdam, The Netherlands; 35 Department of Medical Genetics, Centre for Molecular Medicine and Therapeutics, Child and Family Research Institute, University of British Columbia, Vancouver, British Columbia V5Z 4H4, Canada; 36 Neuroscience, SISSA, Via Bonomea 265, 34136 Trieste, Italy; 37 Department of Neuroscience and Brian Technologies, Italian Istitute of Technology, Via Morego 30, Genova, Italy; 38 Department of Experimental Immunology, World Premier International Immunology Frontier Research Center, Osaka University, Suita, Osaka 565-0871, Japan; 39 RIKEN Center for Life Science Technologies, Wako, Saitama 351-0198, Japan; 40 Melanoma Research Center, The Wistar Institute, Philadelphia, Pennsylvania 19104, USA; 41 German Center for Neurodegenerative Diseases (DZNE)-Tübingen, Otfried Müller Straße 23, 72076 Tübingen, Germany; 42 Laboratory Animal Research Center, Institute of Medical Science, The University of Tokyo, Minato-ku, Tokyo 108-8639, Japan; 43 International Research Center for Infectious Diseases, Institute of Medical Science, The University of Tokyo, Minato-ku, Tokyo 108-8639, Japan; 44 Institute of Health and Biomedical Innovation, Queensland University of Technology, Translational Research Institute, Princess Alexandra Hospital, Brisbane, QLD 4102, Australia; 45 Department of Genetics and Molecular Medicine, King's College London, Guy’s St Thomas Street, London, UK; 46 Centre for Vascular Research, University of New South Wales, Sydney, New South Wales 2052, Australia; 47 Vascular Biology and Translational Research, School of Medical Sciences, University of New South Wales, Sydney, New South Wales 2052, Australia; 48 Division of Cellular Therapy and Division of Stem Cell Signaling, Institute of Medical Science, University of Tokyo, Minato-ku, Tokyo 108-8639, Japan; 49 Harry Perkins Institute of Medical Research, Perth, WA 6009, Australia; 50 Respiratory Medicine, University of Nottingham, Hucknall Road, Nottingham NG5 1PB, UK; 51 Dermatology, School of Medicine Kyungpook National University, Jung-gu, Daegu 41944, Korea; 52 Griffith University, Brisbane, Queensland 4111, Australia; 53 Division of Functional Genomics and Systems Medicine, Research Center for Genomic Medicine, Saitama Medical University, Hidaka, Saitama 350-1241, Japan; 54 Center for Radioisotope Sciences, Tohoku University Graduate School of Medicine, Sendai, Miyagi 980-8575, Japan; 55 Anatomy and Embryology, Leiden University Medical Center, Einthovenweg 20, P.O. Box 9600, 2300 RC Leiden, The Netherlands; 56 Division of Translational Research, Research Center for Genomic Medicine, Saitama Medical University, Hidaka, Saitama 350-1241, Japan; 57 Cell Engineering Division, RIKEN BioResource Center, Tsukuba, Ibaraki 305-0074, Japan; 58 Department of Clinical Molecular Genetics, School of Pharmacy, Tokyo University of Pharmacy and Life Sciences, Hachioji, Tokyo 192-0392, Japan; 59 Department of Bioclinical Informatics, Tohoku Medical Megabank Organization, Tohoku University, Sendai, Miyagi 980-8573, Japan; 60 Department of Biochemistry, Ohu University School of Pharmaceutical Sciences, Koriyama, Fukushima 963-8611 Japan; 61 Insitute for Protein Research, Osaka University, Suita, Osaka 565-0871, Japan; 62 Environmental Epigenetics Program, Biological and Environmental Sciences and Engineering Division, King Abdullah University of Science and Technology (KAUST), Thuwal 23955-6900, Kingdom of Saudi Arabia; 63 University of Delaware, Newark, DE 19716 USA; 64 Hjelt Institute, Department of Forensic Medicine, University of Helsinki, Kytosuontie 11, 003000 Helsinki, Finland; 65 Laboratorio Nazionale CIB, Padriciano, 99 34149, Trieste, Italy; 66 Department of Orthopedic, Trauma and Reconstructive Surgery, Charite Universitatsmedizin Berlin, Charitéplatz 1, 10117 Berlin, German; 67 International Research Center for Medical Sciences (IRCMS), Kumamoto University, Chuo-ku, Kumamoto 860-0811, Japan; 68 Department of Clinical Study, Center for Advanced Medical Innovation, Kyushu University, Higashi-Ku, Fukuoka 812-8582, Japan; 69 Graduate School of Pharmaceutical Sciences, Nagoya University, Nagoya, Aichi 464-8601, Japan; 70 Laboratorio Nazionale del Consorzio Interuniversitario per le Biotecnologie (LNCIB), Padriciano 99, 34149 Trieste, Italy; 71 QIMR Berghofer Medical Research Institute, Brisbane, QLD 4006, Australia; 72 Centre for Stem Cell Systems, Department of Anatomy and Neuroscience, MDHS, University of Melbourne, Melbourne, VIC 3010, Australia; 73 Department of Biochemistry, Nihon University School of Dentistry, Chiyoda-ku, Tokyo 101-8310, Japan; 74 Center for Clinical and Translational Reseach, Kyushu University Hospital, Higashi-Ku, Fukuoka 812-8582, Japan; 75 Telethon Kids Institute, the University of Western Australia, Perth, WA, Australia; 76 Preventive medicine and applied genomics unit, RIKEN Advanced Center for Computing and Communication, Yokohama, Kanagawa 230-0045, Japan; 77 RIKEN Preventive Medicine and Diagnosis Innovation Program, Wako, Saitama 351-0198, Japan

**Keywords:** Systems biology, Cell biology, Computational biology and bioinformatics, Developmental biology, Molecular biology

## Abstract

In the FANTOM5 project, transcription initiation events across the human and mouse genomes were mapped at a single base-pair resolution and their frequencies were monitored by CAGE (Cap Analysis of Gene Expression) coupled with single-molecule sequencing. Approximately three thousands of samples, consisting of a variety of primary cells, tissues, cell lines, and time series samples during cell activation and development, were subjected to a uniform pipeline of CAGE data production. The analysis pipeline started by measuring RNA extracts to assess their quality, and continued to CAGE library production by using a robotic or a manual workflow, single molecule sequencing, and computational processing to generate frequencies of transcription initiation. Resulting data represents the consequence of transcriptional regulation in each analyzed state of mammalian cells. Non-overlapping peaks over the CAGE profiles, approximately 200,000 and 150,000 peaks for the human and mouse genomes, were identified and annotated to provide precise location of known promoters as well as novel ones, and to quantify their activities.

## Background & Summary

Since the completion of the human genome sequencing, role of individual bases has been a central question. An international collaborative effort, FANTOM (Functional ANnoTation Of Mammalian Genome)^1^, delineated a complex landscape of transcribed RNAs (transcriptome) and their regulations. The initial key technology driving the project was to make full-length cDNA clones, representing complete primary structure of transcribed RNA molecules. Sequencing of the full-length cDNA clones uncovered unexpected number of long non-coding RNAs as well as protein coding genes^2–6^. The CAGE (Cap Analysis Gene Expression)^7,8^ protocol, combination with high-throughput sequencing, was developed to monitor frequencies of transcription initiation by determining 5′-end of capped RNAs. The technology was devised to uncover complexity of the transcriptome^4–6^ and elucidate transcriptional regulatory networks by focusing on promoter elements^9–12^. By taking advantage of single molecule sequencer, HeliScopeCAGE was recently developed to provide more sensitive and accurate monitoring of transcription initiation activities^7,8^.

In the fifth round of the FANTOM projects, FANTOM5, the challenge was to capture the transcriptome of many varieties of cell states as possible, to understand the implication of each genomic bases in different contexts. In the first phase of the FANTOM5 project, we targeted cells in steady state, called ‘snapshot’ samples^13^. Our central focus was on human primary cells, while cell lines, tissues and mouse samples were chosen to cover cells inaccessible as isolated human primary samples. The resulting data provided an atlas of promoter and enhancer activities in wide range of cell states^14^, which is a baseline of understanding complex transcriptional regulation. In the second phase, we focused on transitions of cell states by monitoring ‘time course’ samples, such as activations, differentiations, and developments at sequential time points^15^. The monitored activities of promoters and enhancers demonstrated that enhancer activities is the earliest event during dynamic changes of transcriptome. These data sets are being utilized in many other studies inside and outside of the FANTOM5 consortium.

The data production scheme was implemented based on the FANTOM5 collaboration. Sample collection was performed at individual institutes, since specific types of samples require dedicated systems with special expertise or settings, as well as through purchase from commercial sources. RNA quality was firstly examined at the place where the samples were obtained (the first RNA quality check). The CAGE assay pipeline established in RIKEN GeNAS (Genome Network Analysis Support Facility) employed two workflows of HeliScopeCAGE, a manual workflow for samples with small amount of total RNAs^8^ and a robotic workflow for samples with standard requirements^7^. The assay pipeline started with checking RNA quality (the second RNA quality check), which provides a uniform quality assessment of the profiled RNA extracts. The resulting CAGE libraries were sequenced by HeliScope in RIKEN and also in Helicos Biosciences, and the obtained data were processed by the MOIRAI system^16^. Quality of the resulting CAGE profiles was checked with several statistics as well as manual inspection by using the ZENBU browser^17^. Finally CAGE profiles were shared among the consortium for further analysis.

In the course of the two phases focused on ‘snapshot’ and ‘time course’ samples, we profiled 1,816 human and 1,016 mouse samples in total, and obtained approximately four millions of single-molecule reads successfully aligned to the genome per sample on average. Based on frequencies of the observed 5′-ends of individual capped RNA molecules at a single base-pair resolution, we identified 201,802 and 158,966 peaks for human and mouse respectively, where promoters are defined as the sequence immediately upstream of the peaks and frequencies of observed CAGE reads reflect activities of the promoters. All data generated during the course of the project were deposited to a public repository (DDBJ Read Archive, DRA) and/or provided at the FANTOM5 web resource (http://fantom.gsc.riken.jp/5/)^18^. Here we describe the data with the processing details and quality metrics.

## Methods

### Sample collection

Sample collection was performed as described previously^13,15^. Briefly, primary cells were purchased as purified RNAs or frozen cells, or obtained as described previously^19–24^ through collaboration in the consortium. Purchased cells were cultured according to the manufacturer’s instructions and miRNeasy kit (QIAGEN) was used for RNA extraction. Human post mortem tissue RNAs were purchased or obtained through the Dutch Brain bank. Tissues collected through the consortium were snap-frozen in liquid nitrogen, transferred into Lysing Matrix D tubes (MP Biomedicals, Santa Ana, CA) containing chilled Trizol (Gibco), homogenized by FastPrep Homogenizer (Thermo Savant), and centrifuged. miRNeasy kit (QIAGEN) was used for RNA extraction from cultured cell lines as well as frozen cell line stocks.

For the purchased samples, lot or catalogue numbers were recorded where available. Of the collected RNAs, those with more than 1 μg, were measured by Agilent BioAnalyzer (Agilent Technologies, Santa Clara, CA) and Nanodrop spectrophotometer (Thermo Fisher Scientific, Wilmington, DE) to check RIN (RNA integrity) score and the absorbance ratio of A260/A230 and A260/A280. The rest of the samples were directly subjected to the CAGE library production to avoid wasting material. All 2,832 profiled samples are summarized in Table 1.

### Single molecule CAGE and data processing

HeliScopeCAGE libraries were prepared, sequenced, and processed as described previously^13,15^. Most of the RNAs were subjected to the automated HeliScopeCAGE protocol^7^, except for RNAs with less than 1 μg that were subjected to the manual protocol optimized for low quantity RNAs^8^. The resulting libraries were measured by OliGreen fluorescence assay kit (Life Technologies), and sequenced by following the manufacturer’s instructions (LB-016_01, LB-017_01, and LB-001_04 (ref. 13). RNAs extracted from mouse whole body embryo E17.5 (called internal control) were systematically subjected to this workflow, with one per a sequencing run.

**Figure 1:**
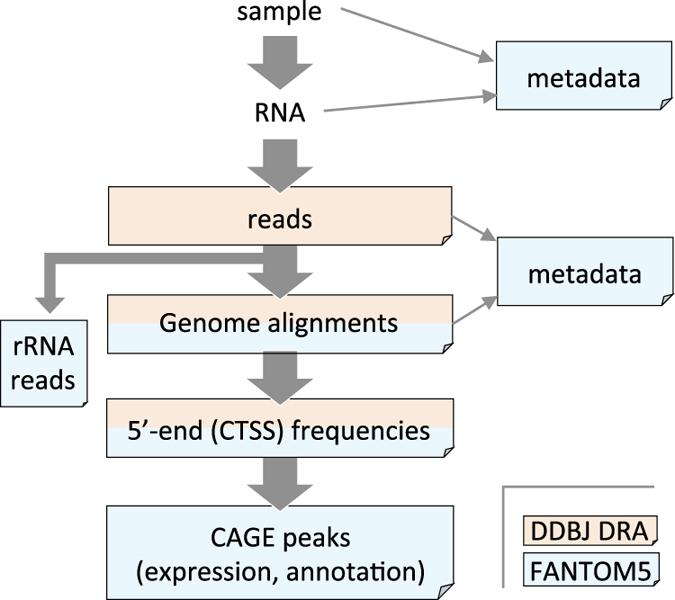
Data processing scheme. Data processing scheme from sample preparation to CAGE peak expression and annotation. Sky blue and beige color indicate locations storing the data, the FANTOM5 data archive (Data Citation 1, Data Citations 10) and in DDBJ Sequence Read Archive (Data Citations 2–9) respectively.

The produced data were processed as previously described^13,15^. Briefly, reads corresponding to ribosomal RNA were removed by using the program rRNAdust (http://fantom.gsc.riken.jp/5/suppl/rRNAdust/), remaining reads were aligned to the reference genome of human and mouse (hg19 or mm9) by using Delve^25^, and alignments with a quality of less than 20 (<99% chance of true) or a sequence identity of less than 85% were discarded. Frequencies of the CAGE read 5′ ends were counted to give a unit of CAGE tag start site (CTSS), a single base-pair on the reference genome. The entire flow of the data is illustrated in Fig. 1, and the number of CAGE profiles (equivalent to CTSS files) is summarized in Table 2.

### Identification of peaks and their annotations

Non-overlapping peaks based on the all CAGE profiles were identified by using DPI (decomposition-based peak identification, https://github.com/hkawaji/dpi1/) method and annotated as previously described^13,15^. A ‘robust’ threshold, for which a peak must include a CTSS with more than 10 read counts and 1 TPM (tags per million) at least one sample, was employed to define a stringent subset of the CAGE peaks. The robust peaks were associated with known transcripts, such as RefSeq^26^, UCSC known gene^27^, GENCODE^28^, Ensembl^29^, and mRNAs (full-length cDNA clones), based on their 5′-end proximity to the peaks. Official gene symbols, Entrez Gene IDs, and protein (UniProt) IDs associated with the transcripts were retrieved and assigned as part of annotation. In addition to these associations, human readable names and descriptions were assigned to each of the CAGE peaks. Peaks were given a name in the form pN@GENE, where GENE indicates gene symbol or transcript name and N indicates the rank in the ranked list of promoter activities for that gene. For example, p1@SPI1 represent the peak with the highest number of observation (that is, read counts) in all of the FANTOM5 CAGE profiles, among the peaks associated with SPI1 gene.

Peak identification with the same method and the same threshold was performed two times; the first was for ‘snapshot’ samples (phase 1), and the second was for the entire samples from both the ‘snapshot’ and ‘time course’ studies (phase 2). We integrated these two peak sets into a hybrid set consisting of all the phase 1 peaks over the robust threshold and a subset of phase 2 peaks that did not overlap with the phase 1 peaks. Annotation of phase1 peaks was used in the hybrid set, called phase 1+2 peaks, which provide a consistent reference in the definition of promoters.

### Quantification of promoter activities

All the obtained CAGE profiles were subjected to the peak identification, even if they have some issues in quality, since all of them still represent independent observations of RNA 5′-ends. However promoter activities (that is, expression levels of CAGE peaks) were quantified only in the samples satisfying the following criteria: RIN score greater than 6, more than 500,000 successfully aligned reads to the genome, and more than 50% of the successful alignments are close to 5′-end of RefSeq gene model, for expression analysis requiring reliable quantification. After discarding a few CAGE profiles of low quality, read counts for individual CTSSs belonging to the same peak were summed up, normalization (or scaling) factors were calculated with RLE (Relative Log Expression)^30^ method by edgeR^31^, and tags per million (that is, counts per million) was computed as expression levels.

The RLE normalization was first performed within the phase 1 samples. The naïve application of this to the entire data sets, consisting of phase 1 and phase 2 samples, might cause inconsistencies in expression levels between the two normalizations. To avoid this, we took the geometric mean of CAGE peak read counts across the phase 1 samples and used it as the reference expression for a normalization factor calculation in the same manner as RLE method. This enabled us to keep the expression levels of phase 1 as they were, and to adjust the expression levels of the phase 2 samples to be comparable^15^.

### Code availability

All software used in this study are publicly available. rRNAdust, for removing ribosomal RNA, is available at http://fantom.gsc.riken.jp/5/suppl/rRNAdust/. Mapping software Delve is available at http://fantom.gsc.riken.jp/5/suppl/delve/. The program to perform DPI, decomposition-based peak identification, method is available at https://github.com/hkawaji/dpi1/.

## Data Records

### Data record 1: Metadata

Two types of metadata are available at figshare and LSDB Archive (Data Citation 1, 10). One is for the samples, including their origins and extracted RNA. The other is for the CAGE assay, including the result of RNA quality check, library production, and post-processing of the CAGE tag sequences. Both of them are described in SDRF (Sample and Data Relationship Format)^32^. Sample metadata for human and mouse are ‘HumanSamples2.0.sdrf.xlsx’ and ‘MouseSamples2.0.sdrf.xlsx’, respectively. The metadata for the CAGE assay are available as ‘*sdrf.txt’.

### Data record 2: CAGE profiles

All of the CAGE sequences, their alignment to the genomes, and CTSS frequencies are available at DDBJ DRA (DDBJ Sequence Read Archive) (Data Citations 2–9). The accession number of each file is summarized in ‘DRA*.txt’ at figshare (Data Citation 1).

### Data record 3: CAGE peaks

Genomic coordinates, annotations and expressions of the CAGE peaks are available as ‘*phase1and2combined_coord.bed.gz’, ‘*phase1and2combined_ann.txt.gz’, and ‘*phase1and2combined_tpm.osc.txt.gz’ respectively at figshare (Data Citation 1). Genomic coordinates are formatted in BED format, and the others are formatted in OSCtable (Order Switchable Column table). The detail of the OSCtable format is available at https://sourceforge.net/projects/osctf/.

## Technical Validation

### RNA quality

**Figure 2:**
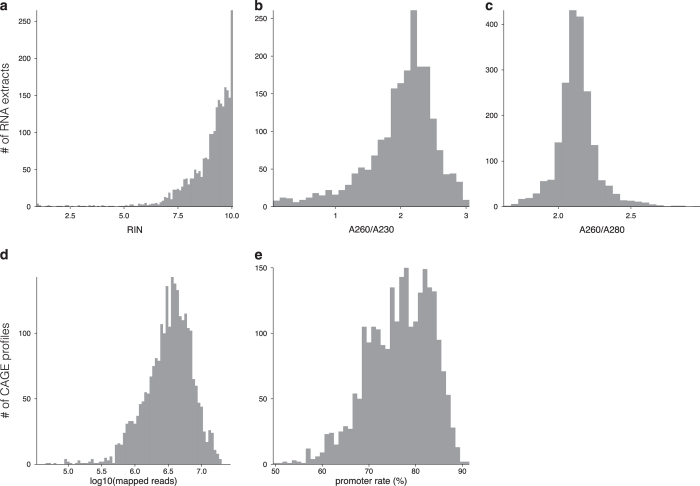
RNA and mapping quality control. Distribution of RIN score (**a**), A260/A230 (**b**), A260/A280 (**c**), mapped reads (**d**), and promoter rate (**e**) for samples used for FANTOM5 expression analysis.

Measured RNA qualities at the second check (that is, immediately before the CAGE library production) are shown in Fig. 2a–c. RNA Integrity Number (RIN) score, measured using an Agilent Bioanalyzer, was 8.96 on average (standard deviation 1.19), absorbance ratio of 260/230 nm (A260/A230) and 260/280 nm (A260/A280) were on average 2.01 (standard deviation 0.53) and 2.13 (standard deviation 0.14) respectively. These figures indicate that the majority of the RNAs were processed in good quality.

### Mapped reads

The number of CAGE reads successfully aligned with the genome and the ratio of CAGE reads hitting conventional promoters are shown in Fig. 2d,e. The average number of mapped reads is 4,208,291 per CAGE profile. Of the 2,522 profiles, 98.3% (2,478) consists of at least 500,000 successfully aligned reads, which was a criterion of profiles used for expression analysis^13^. The average ratio of promoter-hitting reads is 76.5, and 98.6% of the all profiles (2,437/2,472) have more than 50% promoter-hitting rate, which was another criterion of profiles used for expression analysis^13^.

### Sample identity

**Figure 3:**
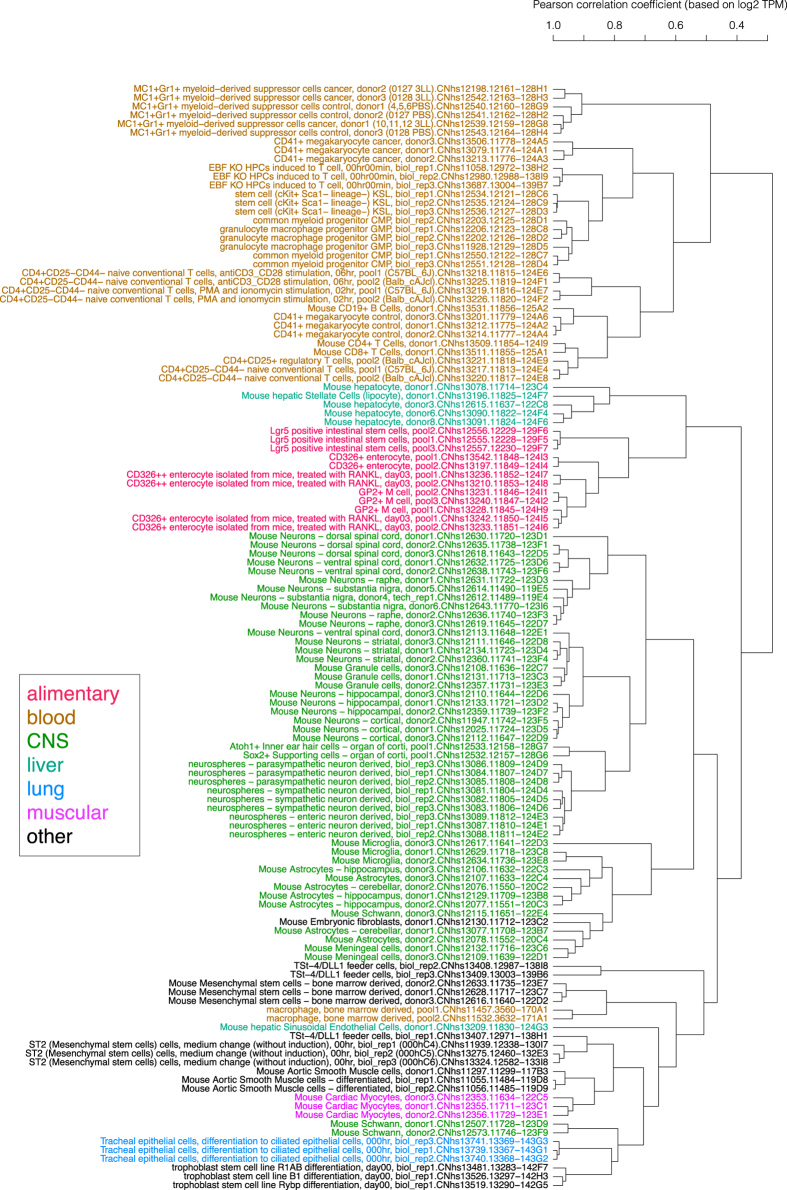
Hierarchical clustering of primary cells. Hierarchical clustering of primary cell samples of mouse based on logarithm of expression (TPM). Color shows anatomical categories of samples.

Hierarchical clustering of the 126 mouse primary cells^13^ within the phase 1 was shown in Fig. 3, and the same clustering of the 571 human primary cells^13^ was in Supplementary Fig. 1. The average linkage method was applied to log-scale expression (TPM) profiles at promoter-level, and sample identities were assessed by expression of marker genes and also by manual inspection of the hierarchical clustering. The figures show that majority of biological replicates belonged to the same branch of the tree, that is, the same cluster, except for samples with a low number of mapped read counts.

## Usage Notes

As well as providing access to individual data files, we also set up a series of interfaces as described in the FANTOM web resource^18,33^. TET (Table Extraction Tool) provides an interface to obtain a subset of data by specifying the desired columns and rows. The BioMart interface^34^, and FANTOM5 SSTAR (Semantic catalog of Samples, Transcription initiation And Regulators) provides the metadata of the profiled samples^35^. The CAGE profile on the genomic axis is visible in ZENBU^17^ with its interactive interface and also in the UCSC genome browser^36^ via track data hub^37^.

## Additional Information

**How to cite this article:** Noguchi, S. *et al.* FANTOM5 CAGE profiles of human and mouse samples. *Sci. Data* 4:170112 doi: 10.1038/sdata.2017.112 (2017).

**Publisher’s note:** Springer Nature remains neutral with regard to jurisdictional claims in published maps and institutional affiliations.

## Supplementary Material



Supplementary Fig. 1

## Figures and Tables

**Table 1:** Summary of FANTOM5 phase 1 and phase 2 samples.

**Sample**	**Phase 1**		**Phase 2**		**Total**
	**Human**	**Mouse**	**Human**	**Mouse**	
Cell lines	259	1	9	0	269
Fractionations	12	0	9	0	21
Primary cells	537	109	24	31	701
Timecourse samples	35	19	748	572	1,374
Tissues	150	237	33	45	465
Quality control samples	0	1	0	1	2
Total	993	367	823	649	2,832

**Table 2:** Sequence files (CTSS files).

**Sample**	**Phase 1**		**Phase 2**		**Total**
	**Human**	**Mouse**	**Human**	**Mouse**	
Cell lines	261	1	10	0	272
Fractionations	12	0	9	0	21
Primary cells	538	110	26	50	724
Timecourse samples	35	20	750	578	1,383
Tissues	152	236	36	45	469
Quality control samples	0	28	0	122	150
Total	998	395	831	795	3,019
