# Supplementary figures and images for: FANTOM5 CAGE profiles of human and mouse samples
Source: Sci Data. 2017 Aug 29;4:170112. doi: 10.1038/sdata.2017.112 (PMC5574368; doi:10.1038/sdata.2017.112)

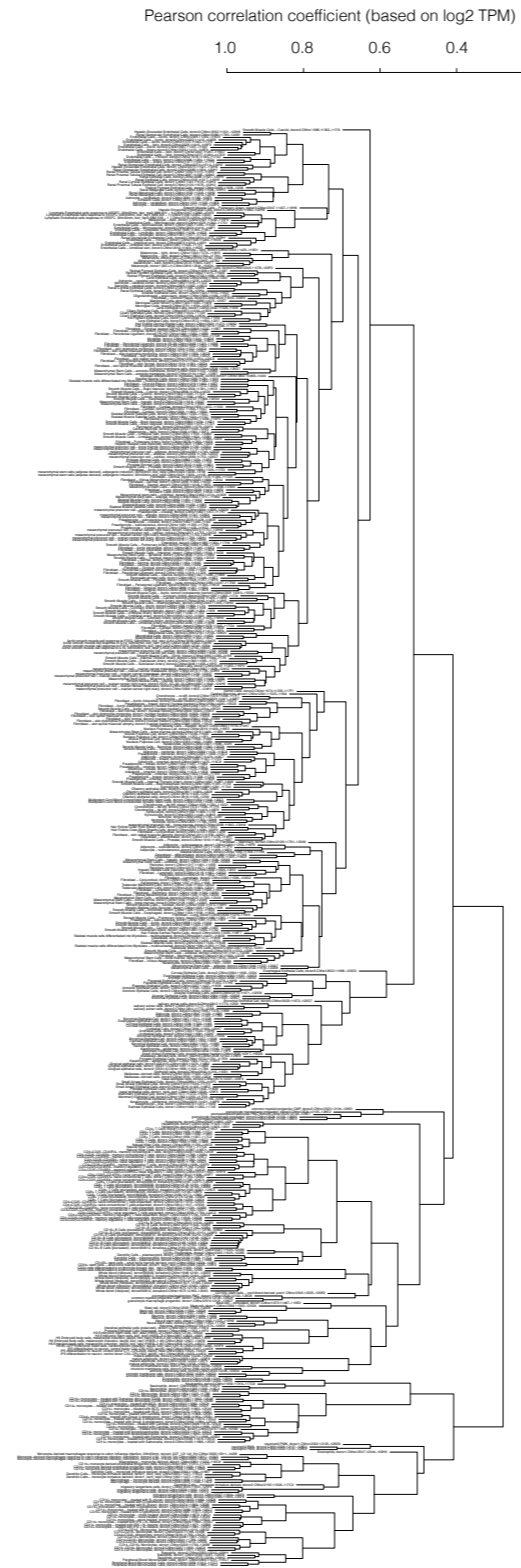

Supplement: Supplementary Fig. 1 [file sdata2017112-s2.pdf]
